# Regional Therapy for Recurrent Metastatic Melanoma Confined to the Extremity: Hyperthermic Isolated Limb Perfusion *vs*. Isolated Limb Infusion

**DOI:** 10.3390/cancers20100043

**Published:** 2010-02-10

**Authors:** Michael Reintgen, Christian Reintgen, Christopher Nobo, Rosemary Giuliano, Steven Shivers, Douglas Reintgen

**Affiliations:** 1 Cutaneous Oncology Program, Lakeland Regional Cancer Center, Lakeland, FL 33804-1057, USA; 2 Department of Surgery, Morton Plant Mease, North Bay Hospital, New Port Richey, FL 34652, USA; E-Mail: rosemarygiuliano@hotmail.com; 3 Moffitt Cancer Center, Tampa, FL 33612, USA; E-Mail: Steven.Shivers@moffitt.org

**Keywords:** metastatic melanoma, extremity, hyperthermia, isolated limb perfusion

## Abstract

Melanoma patients with recurrent disease confined to an extremity can be offered one of two regional therapies that both give high complete response rates. Isolated limb infusion (ILI) is a newer technique performed with catheters and tourniquets that has a reduced potential morbidity, decreased efficacy and does not treat the regional nodal basin. Hyperthermic Isolated Limb Perfusion (HILP) is an open surgical technique that includes removal of the regional nodal basin as part of the surgical procedure. An analysis was performed of the rates of regional nodal disease in this patient population to determine the percentage of patients with stage III metastatic disease to the lymph nodes that would be under treated with the ILI technique. A total of 229 patients underwent a HILP for melanoma with regional lymph node dissection as is our standard between July 1987 and December 2009. Ninty-two of the 229 patients (40%) had metastatic regional nodal disease documented at the time of the HILP procedure. HILP is the only technique that addresses all micrometastatic disease on the extremity.

## 1. Introduction

Malignant melanoma at times recurs on the extremity without any evidence of disease spread beyond the leg or arm. In 1958, Creech and colleagues [1] described a surgical technique in which they would isolate the involved extremity from the remainder of the body and treat the isolated extremity with high temperatures and high doses of chemotherapy. If the disease was indeed confined to the extremity the hyperthermic isolated limb perfusion (HILP) had a 70% chance of controlling the disease. The technique originally described involved an open surgical procedure with a general anesthesia, a 3–4 day stay in the hospital and side effects that range from erythema and lymphedema of the extremity to blood clots and even rarer, limb toxicity that required amputation. 

Recently the Sydney Melanoma Unit has published their series [2] using a minimally invasive technique called an isolated limb infusion (ILI). Originally described by the Sydney group, this procedure involves catheter insertion into the artery and vein of the affected extremity under local anesthesia and perfusion of the extremity with melphalan and dactinomycin. ILI has very small circuit volumes and generates flow rates in the 55–75 mL/min range. In contrast HILP not only uses large circuit volumes but can generate flow rates in the 500–1200 mL/min range. True hyperthermia (synergistic with Melphalan) is obtained with HILP and limb temperatures can reach 40.5 °C while only mild hyperthermia is achieved with the ILI technique. Drug doses are reduced, drug circulation times are shorter, maximum temperatures achieved are less and the environment different for the ILI technique. ILI circulates Melphalan in a hypoxic, acidotic environment while with HILP the oxygenation status of the extremity is maintained, since a heart/lung by-pass machine is used [3]. Because of the above reasons, toxicity associated with the ILI procedure may be less, but the clinical efficacy may also be decreased.

In addition, when a physician is performing an invasive procedure to treat a malignancy, one usually endeavors to treat all known disease. With the usual ILI procedure the catheters are positioned approximately 10–15 cm below the inguinal crease if not more distal. Levels of effective ILI or HILP can be estimated post-operatively with the level of cutaneous erythema that is noted in the post-operative period. Invariably, the regional nodal basin is not treated with the ILI procedure due to catheter positioning being too distal. With HILP, the nodal basin is removed to gain access to the vessels. By doing so a therapeutic node dissection is accomplished when micrometastatic disease is present. The following report is given to determine the percentage of patients that are inadequately treated with the ILI procedure due to microscopic disease in the regional nodal basin at the time of the surgical procedure.

## 2. Material and Methods

The study population consisted of 229 consecutive patients with extremity local/regional recurrence with 55% being female and is described in Table 1. The series was registered on a prospective database at two institutions, Moffitt Cancer Center in Tampa, FL and the Lakeland Regional Cancer Center in Lakeland, FL from 1987–2009. Patients were treated with a single HILP using the drug Melphalan at a does of 1.2 mg/kg for lower extremity lesions and 0.8 mg/kg for the upper extremity.

**Table 1:** Patient populations and results.

	Florida HILP	ACOSOG HILP [9]	Duke HILP [8]	Sydney ILI [2]	MSK ILI [7]	Duke ILI [8]	Multi-Center ILI [6]
Patient Number	229		59	185	30	58	
Complete Response	66%	25%	57%	38%	23%	39%	33%
Partial Response	20%	45%	31%	46%	30%	14%	28%
Stable Disease	10%					10%	
Progressive Disease	4%		12%			46%	
Grade III/IV Toxicity			32%	42%		18%	24%

The HILP procedure has been described previously [4]. Briefly, the patient is given a general anesthesia and the extremity and regional basin are prepped and temperature probes place in 3 areas of the extremity (foot, calf, thigh/hand, forearm and upper arm). The extremity is wrapped in a heating blanket that is maintained with circulating water at a temperature of 40 °C to help heat the extremity and avoid heat loss. For upper extremity HILP, if only a SLN procedure had been performed the axillary artery and vein are isolated and canulated through an axillary approach. If an axillary node dissection had been performed a subclavicular incision is made and the ipsilateral subclavian artery and vein isolated along with a node dissection. Lower extremity HILP are usually performed through the superficial groin if just a SLN procedure had been performed previously, otherwise the approach is through the iliac artery and vein. The perfusion circuit consists of a heart /lung machine with membrane oxygenator and heat exchanger. A Steinmann pin is place in the iliac crest or humoral head and an Esmark is used for a venous tourniquet to eliminate venous collaterals from the extremity and prevent volume loss from the circuit. After adequate heparization, the vein and artery are canulated, the extremity is heated to 40 °C and the patient is perfused for 1 hour with Melphalan. Perfusion pressures are kept below systemic pressures and flow rates of 500–1,200 cc/min are obtained.

The average age of diagnosis of the patient’s melanoma in this series was 60 years and the average age of the population undergoing the hyperthermic isolated limb perfusion (HILP) was 63.8 years. Five percent of the patients had Clark Level II lesions, 17% had Clark Level III melanomas, 62% had Clark Level IV lesions and 16% of the patients had primary melanomas invading in the cutaneous fat. The average tumor thickness of the group was 2.8 mm with a range of 0.15–10.08 mm.

**Figure 1:**
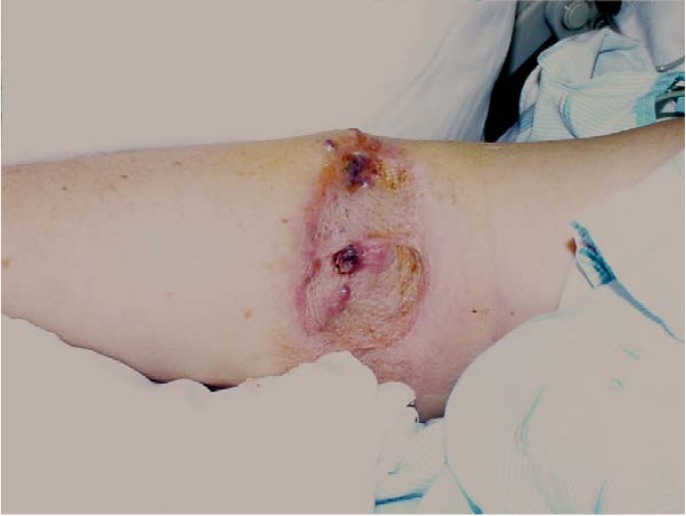
Patients on the trial underwent a HILP for local/regional soft tissue recurrent melanoma confined to the extremity and all had clinically negative regional nodal basins. A typical patient is depicted below with multiple local soft tissue recurrences after a primary melanoma resected and grafted a number of years previously.

HILP was performed in 53% of the cases for local recurrences (Figure 1) while the remainder had the indication of regional soft tissue metastases or satellites. Responses were assessed according to the standard World Health Organization criteria [5]. These define a CR as the disappearance of all measurable disease, determined by two observations less than 4 weeks apart, and a PR as a >50% decrease in total tumor size determined by two observations <4 weeks apart and no appearance of any new lesions or progression of any lesion. Stable disease (SD) was defined as no change or a <50% decrease in tumor size and progressive disease as any increase in size of target lesions. Patients that recurred after the initial HILP were either treated with a repeat HILP or with intra-lesional therapy with BCG or on clinical trial.

## 3. Results

All patients had clinical N0 disease at the time of the HILP and all patients underwent a regional node dissection as part of the procedure to gain access to the vessels for the HILP. If the patients had a previous sentinel lymph node (SLN) biopsy, the regional basin dissection was completed. If the patient had had a previous 1st station complete node dissection, then a second station node dissection (for lower extremities—a hypogastric and iliac dissection and for upper extremities—a subclavian node dissection) was performed again to gain access to the vessels of the extremity for the HILP.

Ninety-two patients (40%) had evidence of microscopic nodal metastases at the time of the HILP. The response rate experienced during the trial was a CR of 66% and a PR of 20%. 10% of the patients had stable disease and 4% of patients had progressive disease. With a mean follow-up of 7 years, 27% of the patient that experienced a CR with the HILP recurred with an average disease free interval from the time of HILP of 12 months.

The morbidity of the HILP was confined to the treated extremity and consisted of erythema to the HILP level and worsening lymphedema in most patients. The lymphedema has to be taken in the context that most of these patients will also have previous primary site surgery, SLN surgery and complete lymph node dissection surgery (both first and second station nodes) along with the HILP. It is unclear what is the contribution to the lymphedema from just the HILP. One patient did require a below knee amputation due to an unrecognized clotting disorder prior to surgery. Another patient did develop leukopenia that required growth factor administration presumably due to leak of the melphalan into the systemic circulation.

## 4. Discussion

A multi-centered group reported recently on the largest US experience with the ILI technique [6]. Of 122 evaluable patients the complete response rate (CR) was 33%, partial response (PR) rate was 28% and there was no response in 39% of the patients (Table 1). Grade III or above toxicity was encountered in 24% of the patients with one amputation that was toxicity related. On multivariate analysis, the variables associated with a better response included smaller limb volumes (p = 0.014) and the use of papaverine in the circuit to induce cutaneous vasodilation (p < 0.001). But the latter variable was associated with higher toxicity. Correction of melphalan dose for IBW did not change response but significantly lowered toxicity. The conclusion of the investigators was that the ILI was a reasonable alternative to HILP in the management of advanced melanoma [1].

John Thompson and the Sydney Melanoma Unit recently reported their 14-year experience with the ILI technique [2]. This group treated 185 patients with a single ILI using melphalan and actinomycin-D. Drug circulation was for 20–30 minutes under mild hyperthermic conditions (38–39 ºC). The overall response rate was 84% with a CR of 38% and a PR of 46%. Median response duration was 13 months, but was noted to be 22 months for patients with a CR. The median survival of the entire group was 38 months and was noted to be significantly increased (53 months) in those patients that experienced a CR (p = 0.005). On multivariate analysis significant factors for a favorable outcome were the achievement of a CR, stage of disease, thickness of the primary, the CO2 level in the circuit and the Wieberdink limb toxicity score of III. Grade III and IV toxicity was seen in 42% of the cases. No grade V toxicity requiring amputation of the extremity was noted. 

There are two other reports in the literature concerning the efficacy of the ILI. The first from Memorial Sloan Kettering reports a complete response (CR) rate of 23% [7]. The second from Duke University documents a 30% CR rate [8].

There is some evidence to suggest that HILP may be more clinically effective than ILI in delivering regional chemotherapy as measured by response rates (Table 1). The response rates listed above do fall in the lower end of the range of those reported in a series of trials of HILP. The American College of Surgeons Oncology group (ACOS-OG) HILP Z0020 trail reported in each arm a 25% CR rate and overall response rates reaching 70% [9]. This higher RR for HILP must be balanced with a suspected increased toxicity with HILP. ILI patients do not have to recover from an open operation and have a lower risk of limb loss and surgeons do not have to take up valuable operating room time requiring a complex surgical set-up and a pump team. Some centers will start with a ILI procedure if available and if no response is obtained with the minimally invasive technique, then a HILP can be offered. Since there is less toxicity with ILI and the technique can effectively treat more distal extremity disease, ILI may be indicated as the initial therapy in patients with other co-morbidities or in patients were nodal disease is not an issue, for instance in patients who have had their regional basin previously dissected. One would anticipate similar response rates for a HILP after an ILI failure with response rates in the 50–70% range reported. Likewise patients have been known to respond to repeat HILP in the scenario of extremity recurrence after an initial HILP, even when the patient is perfused with the same agent [10].

The complication rate with HILP has been reported in the literature. The 30 day mortality rate of over 2,000 patients was 0.6% with death resulting from cardiopulmonary complications or overwhelming sepsis from leukopenia due to leak of Melphalan into the systemic circulation. Major amputations occurred in 0.8% of the patients and most were of lower extremity [11]. Twenty-eight percent of patients develop lymphedema, 11% show some muscular atrophy or fibrosis, 4% have neuropathy, 8% pain and 3% have recurrent infections [12]. Most of the complications noted in the present series were due to worsening lymphedema associated with the nodal dissection in combination with the HILP.

This study shows that with ILI procedures, 40% of the patient population will have untreated disease in the nodal basin. Some have suggested that the presence of iliac, hypogastric or subclavian nodal disease is a sign of systemic disease and patients should be staged as Stage IV disease. However the 6^th^ edition of the AJCC staging manual stages these patients as advanced Stage III disease. Certainly anatomically the latter makes sense, since the superficial groin lymphatics flow directed into the deeper lymphatics of the pelvis through Cloquet’s node and anatomically the same is true for the relationship between the axillary and subclavian nodal basins. 

What is the mechanism that these nodal metastases may exist after the initial treatment of the primary melanoma. Even to this day, some patients are not having their nodal basins addressed at the time of the radical resection of the primary melanoma. Depending on tumor thickness, a percentage of these patients will have microscopic nodal disease left behind that will become apparent with the appearance of intransit metastases and a nodal dissection with the HILP. Some patients will not undergo a CLND after a positive SLN biopsy despite the fact that approximately 15% of these patients will have microscopic disease beyond the SLN. Nodal metastases will then be found with any subsequent nodal dissection and HILP. In patients with a negative SLN biopsy with the initial cancer operation, about 5% will have a false negative result. These patients will have nodal metastases at the time of HILP. In addition, the local recurrence metastases or intransit metastases that give the patient the indication for a HILP may metastasize to the regional nodal basin in a different distribution than metastases from the primary melanoma. Certainly this different pattern of metastases from metastatic lesions is well documented in the lymphatic mapping literature and allows for lymphatic mapping from the metastatic focus despite the patient having a previous mapping from the primary site. Finally, even if a patient has an initial positive SLN followed by CLND of the involved basin, second station metastases may occur either from direct drainage from the primary site or from the intransit metastases. Thus, nodal disease may occur in a variety of mechanisms in this scenario.

A large percentage of patients, those with microscopic nodal disease are undertreated with a ILI. Combining ILI with a systemic therapy would seem to more fully solve the problem, but systemic therapy for advanced melanoma is associated with low response rates. The advantage of the HILP procedure is that the regional basin is addressed and treated with what amounts to a therapeutic lymph node dissection.

The delivery of regional chemotherapy to an isolated limb continues to be an important treatment for those patients with recurrent disease confined to the extremity. HILP may be associated with more side-effects and a longer recovery time for the patient, but the technique has the best chance of being able to address all micrometastatic disease in the patient with advanced Stage III melanoma.

## 5. Conclusion

ILI is a new technique touted as a regional therapy for patients with recurrent melanoma confined to an extremity. Proponents of the procedure state that efficacy is better than what can be achieved with systemic therapy and in comparison to the HILP procedure, has less morbidity. However, our data shows that 40% of patients may be inadequately treated with the ILI procedure since it does not effectively treat the regional nodal basin. In patients with more distal recurrent extremity melanoma and the regional basin is not an issue (those with previous nodal dissections) or those patients with significant co-morbidities, ILI may be the initial procedure of choice.
